# Synchrony and asynchrony between an epigenetic clock and developmental timing

**DOI:** 10.1038/s41598-019-39919-3

**Published:** 2019-03-06

**Authors:** Akina Hoshino, Steve Horvath, Akshayalakshmi Sridhar, Alex Chitsazan, Thomas A. Reh

**Affiliations:** 1 Department of Biological Structure, University of Washington, Seattle, WA 98195 USA; 2 Human Genetics and Biostatistics, David Geffen School of Medicine, University of California Los Angeles, Gonda Research Center, Los Angeles, CA 90095-7088 USA; 3 Department of Biochemistry, University of Washington, Seattle, WA 98195 USA

## Abstract

Epigenetic changes have been used to estimate chronological age across the lifespan, and some studies suggest that epigenetic “aging” clocks may already operate in developing tissue. To better understand the relationship between developmental stage and epigenetic age, we utilized the highly regular sequence of development found in the mammalian neural retina and a well-established epigenetic aging clock based on DNA methylation. Our results demonstrate that the epigenetic age of fetal retina is highly correlated with chronological age. We further establish that epigenetic aging progresses normally *in vitro*, suggesting that epigenetic aging is a property of individual tissues. This correlation is also retained in stem cell-derived retinal organoids, but is accelerated in individuals with Down syndrome, a progeroid-like condition. Overall, our results suggest that epigenetic aging begins as early as a few weeks post-conception, in fetal tissues, and the mechanisms underlying the phenomenon of epigenetic aging might be studied in developing organs.

## Introduction

Human and mouse studies have shown that DNA methylation-based biomarkers can provide a way to measure chronological age across the entire age spectrum and in all tissues, despite the fact that patterns of DNA methylation vary considerably across tissues^1–4^. Remarkably, so-called DNA methylation epigenetic clocks provide highly accurate age estimators for individuals from 0 to 100 years old^3,5,6^. The Horvath clock is based on the weighted average of 353 CpGs, whereby methylation of 193 CpGs is positively correlated with age, while methylation of the other 160 CpGs is negatively correlated with age^7^. This DNAm clock has also been used to investigate cases of epigenetic age acceleration, where the individual might be aging more rapidly than their chronological age. In particular, Horvath and colleagues have shown that the age-related phenotypes observed in some progeroid syndromes (e.g., Werner syndrome, Hutchinson Gilford Progeria Syndrome; Down syndrome) correlates with accelerated epigenetic aging^8,9^. While many studies have thus validated the DNAm clock in many different tissues after birth, a recent study shows this clock might also apply prenatally. Analysis of DNA extracted from cord blood from neonates demonstrated that gestational age can be reliably predicted between 24 and 44 weeks^10^. These results are consistent with results from induced pluripotent stem cells (iPSCs) that show they are considerably younger than the cells they were derived from^3,11^.

The studies described above thus motivated us to evaluate the range in human fetal development over which the Horvath’s epigenetic clock is applicable. The use of iPSCs in age-related disease modeling, show biomarkers of age-acceleration before the onset of age-related manifestations of the disease^12–17^. Evidence from many labs for both mouse and human indicates that developmental timing is conserved in tissues derived *in vitro* from pluripotent stem cells, such as cerebral and retinal organoids^18–22^, but it is not known whether tissues derived from iPSCs follow the same epigenetic clock as their corresponding fetal tissues.

To investigate the ability to estimate the age in embryonic or fetal tissue, we utilized the highly regular sequence of development found in the mammalian neural retina^23,24^. During retinal development, the six main types of neurons are generated in a sequence that is largely conserved across vertebrates. Ganglion cells, horizontal cells, and cone photoreceptors are produced by progenitors early in retinogenesis, while most rod photoreceptors, amacrine cells, and bipolar cells are generated in the second half of the neurogenesis period^23,24^. This well-defined sequence of cell generation in the vertebrate retinas is analogous to that which occurs in other developing neural tissues, such as the Drosophila neuroblast lineage^25^ and the cerebral cortical laminar-specification^26^, and provides an excellent model system to study developmental timing. Thus, this study explores the link between developmental and chronological age using the human retina as a model. Our results show for the first time that (1) the epigenetic clock extends back to at least 8 weeks of gestational age, and possibly to conception, (2) the epigenetic clock continues to tick in explant cultures, thus providing potential to explore the mechanisms underlying DNAm epigenetic age, (3) that organoids derived from ESCs also show a correlation between their DNAm age and their chronological age from the onset of the differentiation protocol, and (4) the progeroid-like disorder, Down syndrome, leads to accelerated DNAm age in fetal stages, but does not cause dramatic correlated acceleration of developmental stage, suggesting that the epigenetic clock and the developmental clock are likely independent. Overall, this study provides both an independent way to measure developmental time and establishes the human retina as a potential experimental model to study epigenetic aging *in vitro*.

## Results

**Figure 1:**
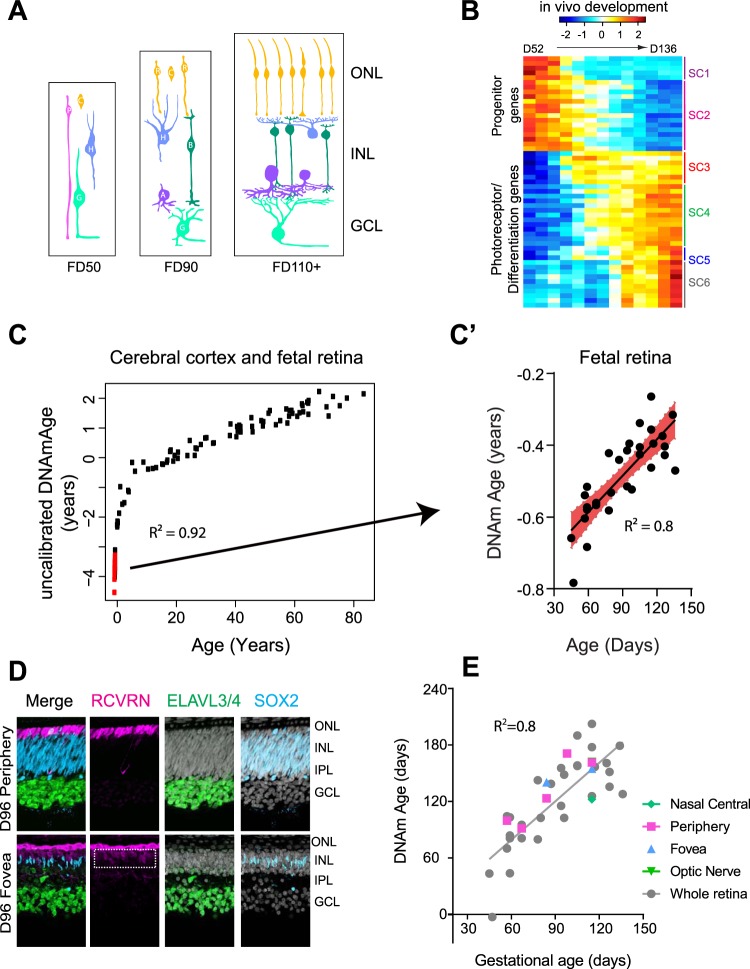
(**A**) Development of the retina proceeds in a regular order of cell addition, with the progenitors (P, pink) first making the early cell types, ganglion cells (G, green), the horizontal cells (H, blue), and then the cones (C, orange), followed by a later phase of histogenesis where the late cell types are generated, including the amacrine cells (A, purple), the rod photoreceptors (R, orange), the bipolar cells (B, dark green), and the Müller glia (not shown). Cell genesis is followed by a regular sequence of differentiation events during which the cells elaborate dendrites and make synapses with one another. (**B**) Bulk RNAseq of fetal retina was organized into six superclusters to highlight gene expression trends during retinogenesis (data re-plotted from Hoshino *et al*.^27^). (**C**) Chronological age (x-axis) versus epigenetic age (y-axis) of cerebral cortex and fetal retina. C’. Expanded view of the correlation between DNA mAge and gestational age of fetal retinal samples. Slope = 1.235 ± 0.1676, R square = 0.8; NOTE: that the time axes are not the same between C and C’. (**D**). Human fetal retina exhibits characteristic central to periphery gradient, where the fovea is more accelerated than the periphery (P) at the same age. The periphery is mostly comprised of progenitors (SOX2, cyan), photoreceptors (RCVRN, magenta) and ganglion/early amacrine cells (ELAVL3/4,green) in a D96 fetal retina while fovea exhibits box-shaped cones and additional RCVRN-positive bipolars (dashed rectangle), amacrine (ELAVL3/4 in the INL) and Müller glia markers (SOX2 in INL) indicative of accelerated retinal development (DAPI in white) E. Epigenetic age remains conserved across different regions of the retina, despite their developmental differences. (ONL = Outer nuclear layer, INL = Inner nuclear layer, GCL = Ganglion cell layer, IPL: Inner plexiform layer; SC = supercluster). R^2^ in E = 0.8.

During retinal development, retinal neurons are specified in a stepwise, temporally-restricted sequence. In the fetal human retina, the first wave of neurogenesis generates ganglion cells, cones and horizontal cells, resulting in a 3-layered retina by day 50 (Fig. 1A, FD50). By 90 days, the second wave of neurogenesis is initiated, leading to the birth of bipolar, amacrine, rod photoreceptors, and Müller glia (FD90). Following the generation of retinal cells, retinal circuits continue to mature (FD110+). We have previously described these main epochs of human fetal development using RNAseq (Hoshino *et al*.^27^) and found approximately 3000 developmentally regulated genes organized into 6 superclusters that highlight meaningful gene expression patterns across the ages D52 to D136 (Fig. 1B).

We analyzed the DNA methylation patterns from the same developmental ages as the RNAseq study to evaluate DNA methylation patterns. These analyses utilized the Horvath epigenetic clock that estimates age based on the DNA methylation (DNAm) levels of 353 CpGs. The resulting age estimate is referred to as DNAm age, and is in units of years, and negative and positive values of epigenetic age correspond to prenatal samples and postnatal samples, respectively. Our data demonstrates that the epigenetic clock is indeed maintained in the retina, and we found a strong correlation (r = 0.80, p < 0.0001, Fig. 1C,C’; Supplemental Fig. 1) between the epigenetic age and chronological age in the fetal retina. This degree of correlation is typical of that which we observe in human adult tissues with the Horvath clock; however, we did not see a similar correlation with other types of DNAm clocks^5^ (Supplemental Fig. 2). The more rapid change in DNAm age in the fetal retinal samples (Fig. 1C red dots) and fetal cortical samples indicates that the epigenetic clock “ticks” more rapidly in the fetal samples than in mature tissues (Supplemental Fig. 1B). The steeper clock rate in the fetal samples allows for better resolution in developing tissues than what is possible in adult tissue, and as a result, one can detect differences in epigenetic age in a few weeks, as opposed to a few years in adult tissues.

To better understand the relationship between the epigenetic clock and the retinal developmental clock that controls the rate of neurogenesis and timing of gene expression, we relied on the developmental gradient present in the fetal retina. Within the human retina, there exists a central-to-periphery gradient, where the center (Central (C) or macula (M)) is developmentally accelerated by several weeks when compared with the periphery (P). RNAseq data (Hoshino *et al*.^27^) demonstrates that the central retina turns off progenitor genes earlier than the periphery at the same age (Superclusters 1&2), and consecutively turns on differentiating photoreceptor cell markers faster (Supercluster 4) (Supplemental Fig. 3). This developmental gradient is particularly noticeable in the generation and morphology of later born cell types, as shown in a D96 retina (Fig. 1D). The peripheral retina at this stage is comprised of photoreceptors (RCVRN, magenta) in the outer layers, followed by progenitors (SOX2, cyan) and ganglion cell layer (ELAVL3/4, green). In comparison, the fovea is developmentally accelerated, as evidenced by the RCVRN positive cones that have a box-shape morphology and RCVRN-positive bipolar cells (dashed rectangle box). In addition to ganglion cells, the fovea also has a distinct population of amacrine cells in the INL (ELAVL3/4, green) and developing Müller glial cells (SOX2, cyan). Interestingly, comparisons of the epigenetic clock from different regions of the retina revealed no major differences; different parts of the retina followed the same trend as the whole retina and maintained their epigenetic age, even though central retina is developmentally accelerated compared to peripheral retinal regions (Fig. 1E). This result suggests that the developmental clock and the epigenetic clock might be controlled independently.

**Figure 2:**
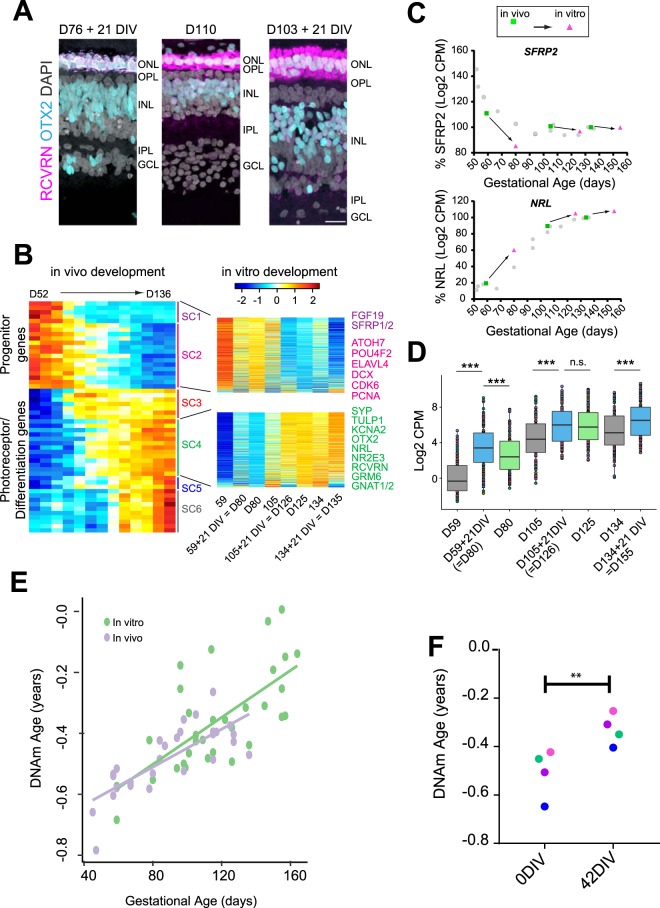
(**A**) D76 and D103 retinal explants maintained for 21 DIV were compared to a D110 fetal retina processed acutely; immunostaining with OTX2 (cyan) and RCVRN (magenta) to label photoreceptors and bipolar cells and DAPI (white, all cells). (**B**) (Left) Heatmap of RNAseq data of human retinas from D52 to D136 (from Hoshino *et al*.^27^) showing all the Superclusters (SCs) of genes based on their developmental expression profiles. Heatmaps of SC1 and SC2 (top) and SC4 (bottom) gene expression of retinal pairs from D59, D105, and D134, with one retina taken for RNAseq immediately postmortem and the other after 21 days *in vitro*. (**C**) Plots for SFRP2 (a progenitor gene) and NRL (a rod photoreceptor gene) for each pair to show that the trends for the expression levels of these genes *in vitro* (pink symbols) is similar to that found in normal *in vivo* development (green symbols for paired retina, gray symbols for all other retinas). (**D**) Boxplot of expression level of the top highly expressed 100 genes from SC4 for the retinal pair data plotted in B, (p < 0.0001, two-sided paired t-test, NS = 0.9). (**E**) Retinas maintained *in vitro* for 42 days (blue) follow the same progression in epigenetic DNAm ages (y-axis) as those of uncultured retinas (gray dots). F. Retinal pairs (matched colors), one analyzed immediately post-mortem and the other after 42 days *in vitro*, show that the epigenetic DNAm age (y-axis) continues to progress *in vitro* (p-value <0.005, one-sided paired t-test).

In order to test whether the epigenetic clock continues to progress when isolated from the rest of the organism, epigenetic clock analyses were performed on explant cultures of fetal retinas from various stages of development. Explants were maintained for 3 to 6 weeks and the rate of development and the epigenetic clock was evaluated by immunofluorescence/RNAseq, and DNA methylation respectively. Figure 2A shows examples of retinas that were either freshly harvested (*in vivo*) or maintained in explant culture for 3 weeks. With the exception of retinal ganglion cells, which do not survive well in explant cultures, other retinal cell types appear to differentiate in approximately normal numbers and with appropriate timing of the onset of many cell-type specific marker genes (Photoreceptors and bipolar cells marked by RCVRN/OTX2 expression in magenta/cyan respectively; Supplemental Fig. 4). More systematic evaluation of retinal development *in vitro* was possible with RNAseq (Fig. 2B–D). Using the superclusters that capture developmental progression in the human retina^27^, and comparing *in vitro* and age-matched *in vivo* development samples, we observed that the explant cultures closely followed normal retinal development (Fig. 2B). Examples of key developmental genes, SFRP2 (an early progenitor gene, Fig. 2C, upper) and NRL (an early rod photoreceptor-specific marker, Fig. 2C, lower) show similar developmental timing between the explant cultures (pink symbols) and the *in vivo* retinas (green symbols for matched pair and gray symbols for all fetal retinal *in vivo*). Figure 2D shows boxplots for the top 100 genes in Supercluster 4, demonstrating developmental progression between retinal pairs when one was taken for RNAseq acutely and the other maintained *in vitro* for 3 weeks. In all cases, gene expression shows appropriate developmental progression *in vitro*.

Given the fact that normal developmental timing is well conserved in human fetal retinal explants, we next determined if epigenetic clock continues to operate normally *in vitro*. For these studies, we cultured 10 retinas, and analysis of their DNAm age (Fig. 2E, blue) showed they progressed at approximately normal rates when compared with the acutely isolated samples (Fig. 2E, gray). We also obtained four pairs of retinas, each pair from a single fetus, and extracted DNA from one eye immediately and the other eye after it was maintained in explant culture for 6 weeks (Fig. 2F). For each pair, the epigenetic clock continued at the normal rate *in vitro*, advancing to the appropriate chronological age as that of a similarly aged *in vivo* retina (Fig. 2F, p < 0.005). These results indicate that the synchrony between the developmental and aging clock is maintained outside the body, and suggest that the aging clock is an independent property of the tissue.

**Figure 3:**
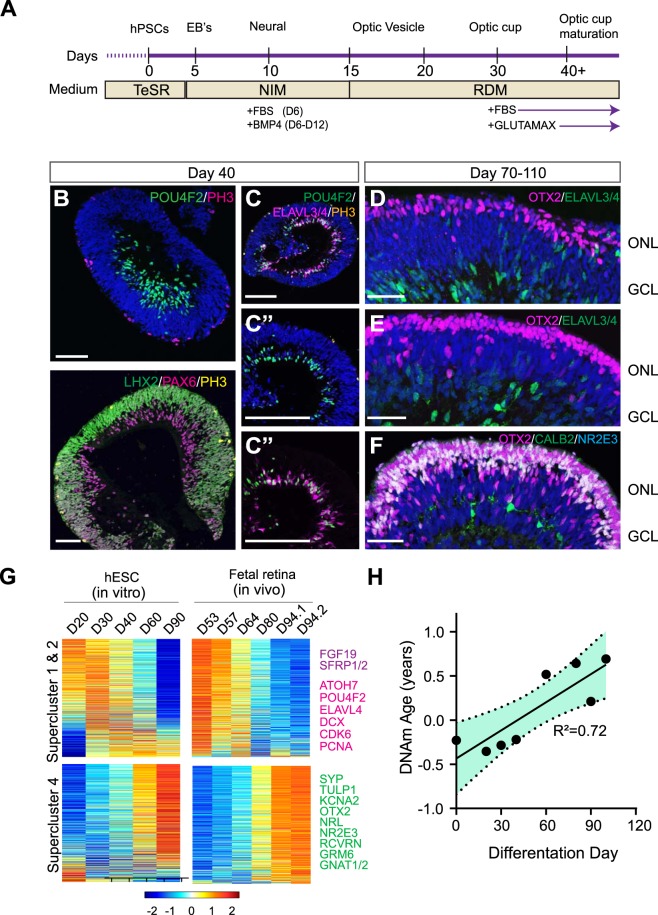
(**A**) hPSCs were directed to generate retinal organoids in a step wise manner as previously described^31,32,55,57^. (**B**,**C**). By 40 days, ganglion cells were readily identified by the expression of POU4F2 and PAX6 within inner layers of organoids, and remained distinct from retinal progenitors (LHX2). Early organoids expressed ELAVL3/4 and POU4F2 in a ring-like manner within basal layers of organoids, (C, 200 µm, C’-C” 50 µm), while mitotic marker PH3 was localized towards the apical surface of organoids. (**D**–**F**). By 70 days, OTX2 positive photoreceptors were seen toward the apical surface of organoids (**D**), and became increasingly abundant by Day 80 (**E**). By 110 days, multiple layers of photoreceptors were visible on the apical surface, and further differentiation yielded NR2E3 positive rod photoreceptors (**F**). (**G**) Bulk RNAseq analysis demonstrate that organoids mimic fetal retina gene expression patterns at comparative stages. Supercluster 1 and 2 genes marking retinal progenitor, cell cycle, and ganglion cells genes decrease in expression while supercluster 4 genes indicative of later-born retinal cell types increase expression at later timepoints. (**H**) DNAm (years) age plotted against the day since the differentiation was initiated (in days) (p < 0.0073).

To further test whether the aging clock is an independent property of the tissue, we asked if cells that develop completely *in vitro* would also follow the epigenetic clock, such as stem cell-derived retinal organoids. Over the last decade, several groups including ours, have demonstrated the ability of pluripotent stem cells to mimic specific stages of retinogenesis, and generate multilayered, stratified structures called retinal organoids^16,18,28–30^. We derived retinal organoids from stem cells using the established protocols^31,32^ (Fig. 3A), and analyzed them for developmental and aging state. Similar to the fetal retina, ganglion cells are the earliest cell type expressed in the basal layers of organoid at 40 days (Fig. 3B,B’, POU4F2 in green, higher PAX6 expression in magenta). Ganglion cells exhibited a ring-like organization within the basal layers of the organoids, and expressed markers for POU4F2 and ELAVL3/4. Following ganglion cell expression, cone photoreceptors were the next cell type to develop in the outer layers of organoids (Fig. 3D–F, OTX2, magenta). Multiple layers of photoreceptors were visible by Day 80, and further differentiation yielded early rod photoreceptors (Fig. 3F, NR2E3 in cyan- looks white in figure because of overlap.) Organoids approximately follow the developmental clock of the fetal retina; however, it is difficult to exactly compare them with a resolution of less than 3 weeks, since there is a large central-to-peripheral developmental gradient across the retina and it is not clear what the retinal position the organoids represent. Moreover, the cellular composition of the organoids is not exactly the same as the fetal retina, since ganglion cells do not survive well *in vitro*. Nevertheless, RNAseq cluster analysis of organoids from Day 20 to Day 90 shows that expression of Supercluster 1&2 genes decreased while expression of Supercluster 4 genes increased with time (Fig. 3G).

Following RNAseq analyses, DNA from the same samples was utilized for epigenetic clock analyses. We found that the DNAm age of the retinal organoids increased with age since the onset of differentiation (Fig. 3H). Interestingly, the epigenetic clock is somewhat accelerated in organoids. For example, samples from 60–100 days of differentiation had a DNAm age comparable to nearly one year old postnatal tissue samples. Nevertheless, their DNAm age remained highly correlated with chronological age after their exit from the pluripotent stage.

**Figure 4:**
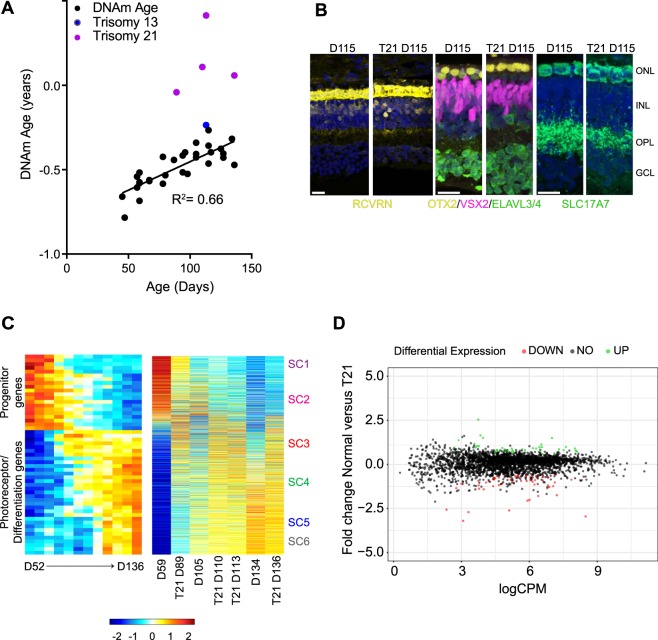
(**A**) DNAm age (years) plotted against chronological age for Trisomy 21 (T21, Down syndrome, magenta) and Trisomy 13 (blue). The T21 retinas were significantly accelerated when compared to the normal fetal retina (p value < 0.0001) (**B**) Immunostaining of Down syndrome and normal fetal retinas for photoreceptors (RCVRN, OTX2) and bipolar cells (VSX2) amacrine cells (ELAVL3/4) and synapses (VGLUT/SLC17A7) taken from D115 age-matched samples at equivalent eccentricity (scale = 20 µm). (**C**) Heatmaps showing bulk RNAseq of Down syndrome and normal fetal retinas (right) compared with supercluster genes from Hoshino *et al*.^27^ (left). D. MA plot showing the similarity of gene expression between WT and Trisomy RNAseq with all ages combined and significantly (p < 0.05) upregulated genes (green) and significantly (p < 0.05) downregulated genes (red).

So far, our data demonstrates that the epigenetic clock of fetal retinal tissue is usually synchronized with development age. Previous analyses have demonstrated accelerated aging in Down syndrome, from both clinical manifestations and the epigenetic methylation clock. Using blood leukocytes, whole blood, and post-mortem brain regions, Horvath *et al*. 2015 reported a highly significant age acceleration of 4–11 years in the Down syndrome cases^33^. Given that the epigenetic age can already be measured in fetal retinal samples, we asked whether epigenetic clock acceleration in Down syndrome might already be measurable in fetal development. We measured the epigenetic age in four Trisomy 21 samples and one Trisomy 13 sample, and we found a significant age acceleration in the four Trisomy 21 samples, but not the Trisomy 13 sample (Fig. 4A, p < 0.0001). Interestingly, the acceleration of the epigenetic clock in these samples was not accompanied by an acceleration of their developmental clock: immunofluorescent labeling of retinal sections show similar patterns of cell differentiation in the Down syndrome cases as in the age-matched normal fetal retinas (Fig. 4B). These images show comparisons between Down syndrome and normal fetal retinas for photoreceptors (RCVRN, OTX2) and bipolar cells (VSX2) amacrine cells (ELAVL3/4) and synapses (VGLUT/SLC17A7). If there was accelerated development in the Down syndrome retinas, we would expect to see a greater number of VSX2+ bipolar cells, more mature photoreceptors (RCVRN) and an increase in VGLUT/SLC17A7 labeling, but we do not see these changes. We further analyzed the developmental state of the Down syndrome retinas using RNAseq (Fig. 4C,D), and our analysis shows similar patterns of gene expression in the ~3000 supercluster genes between Down syndrome and normal retinas (Fig. 4C, Supplemental Fig. 5B–D). The heatmaps shown that the overall trends in the Down syndrome samples follow those of the normal fetal retinas (Fig. 4C); however, there are some differences, and the MA plot (Fig. 4D) shows that 95 of the supercluster genes show significant differences (3% of total supercluster genes; Extended Data Table 3). Of these 95 genes, 8 were on Chr21 and these all were increased in the Down syndrome samples (Supplemental Fig. 5A and Extended Data Table 3).

## Discussion

In the past five years a number of reports have shown that distinct epigenetic changes occur during the process of aging, and these changes show a high degree of correlation with chronological age^4,34,35^. The pan tissue clock by Horvath (2013) accurately estimates age in children^3,4^, and is applicable to DNA from all tissues and nucleated cells, with widely varying cellular composition, from birth to old age. Although most of the previous studies that have shown a high degree of correlation between DNAm age and chronological age have been in mature tissues, two previous studies have shown that the same aging clock might also apply to developing tissues^10,36^. In this report, we have confirmed these earlier studies that used cord blood in neonates and fetal brain, extending this correlation to fetal retinal tissues as well. We also find that the resolution of the clock in fetal tissue is much better than in the adult tissues, like the earlier report in fetal brain. Differences in tissue age of just 4–6 weeks can be discriminated by the epigenetic clock in fetal tissue, while adult tissue can only be discriminated with this clock within 2–4 years. Interestingly, the epigenetic clock resolution is intermediate in infants and children, consistent with a general slowing in the clock rate with maturation. Thus, together with these previous reports, it appears that the epigenetic clock age operates across lifespan, including fetal stages of human development.

Having established that the epigenetic clock can be used to study age in developing tissue, we went on to study the relationship between the epigenetic and the developmental clock that controls the sequence and relative time of events in developing tissues. We have found that the epigenetic clock continues to advance in explant cultures of fetal retina and shows a similar relationship with developmental stage and chronological age in organoids derived from pluripotent stem cells. However, we also find that the developmental clock and the epigenetic clock are not always in synchrony: (1) developmental gradients across the retina are not reflected in DNAm age, (2) organoids are significantly accelerated in DNAm age, despite their similar developmental time with fetal retinas, and (3) the epigenetic clock is significantly accelerated in fetal retina from Down syndrome samples, though the developmental clock does not show major changes and remains correlated with gestational age.

An interesting feature of the epigenetic clock is that it is not restricted to a specific tissue. Previous studies have shown that this is a tissue independent correlation, with a similar age estimate from the same CpGs regardless of tissue type, including brain, skin, kidney, colon, lung, etc^7^. Our study extends this correlation to the neural retina, and further supports the hypothesis that the age is present in all cell types within the tissue. The range of ages of retinal development included in this study encompass major changes in cellular composition; in the early fetal stages, the retina is primarily composed of retinal progenitors and ganglion cells, while at the later time points in our study, the retina is largely composed of amacrine cells and photoreceptors (Fig. 1A). Nevertheless, the epigenetic clock appears to be not dependent on the relative percentages of the different cell types. Additionally, the retina shows major stage-dependent changes in the DNA methylation status of CpGs at the promoters of differentially expressed gene promoters^37^, like other developing tissues, but the DNAm age does not depend on whether the 353 CpGs are in gene promoters of genes expressed in the tissue. Fewer than 10% of the 353 CpGs that comprise the epigenetic clock are in promoters of genes differentially expressed in developing retina^37^. This is similar to fetal brain^38^, where large changes in the methylome accompany a similar range of fetal developmental stages, but the 353 CpGs of the epigenetic clock correlate with gestational age rather than with tissue composition or developmental gene expression. Thus, the developmental changes in methylation occur in parallel with the epigenetic clock, and appear to be reflected in all cells, even transient progenitor populations.

Despite the strong correlation with gestational age and DNAm age in the fetal samples, the epigenetic clock and the developmental clock are not always in synchrony. The development of mammals shows a tight correlation between the gestational age and developmental stage, and staging fetal and embryonic tissues depends on this correlation. However, we took advantage of the natural developmental gradient in the retina to explore the degree of synchrony between the developmental clock and the epigenetic clock. In the human retina there is a pronounced central-to-peripheral gradient in the relative timing of developmental events. For example, the onset of rhodopsin expression in rod photoreceptor in central retina occurs at FD105, but not until FD 250–280 in the far periphery, a difference of over 4 months^39^. The epigenetic clock, however, shows that the central and peripheral regions of the retina have a similar DNAm age, despite this difference in developmental stage (Fig. 1E). This result suggests that the DNAm age is a large-scale property of the tissue, unaffected by regional differences in developmental timing within the tissue. A second example of disconnect between the epigenetic and developmental clock is shown by the analysis of the Down syndrome fetal retinas (Fig. 4). These retinas are significantly older by the DNAm age than their counterpart control retinas, by nearly a 3-fold change. However, this epigenetic clock acceleration does not cause a major change in the developmental clock. We saw few differences in the expression of the 3000 supercluster genes that encompass the major developmental epochs in human retina^27^, between the Down syndrome retinas and age-matched control samples. Taken together, these results suggest that even though the DNAm aging clock and the developmental stage typically correlate with the gestational age, these clocks can be decoupled in some cases and likely represent two independent timing mechanisms.

Another interesting feature of the epigenetic clock revealed by our experiments is that this clock continues to correlate with gestational age *in vitro*. Previous studies in our lab and other have shown that fetal retinas can be maintained as explants *in vitro* for weeks, and that they continue to follow their normal course of development^40^. In this study, we analyzed retinal explant cultures and found that in addition to following their normal developmental clock, they also advance in DNAm age at the same rate as they would have *in vivo*. To our knowledge, this is the first demonstration of an epigenetic aging clock continuing to tick *in vitro* and suggests that the clock depends on a fundamental process in the cells that is not altered by the artificial environment of the organ culture. Moreover, the mechanisms driving the Horvath epigenetic clock appear to be a property of cells and tissues, rather than the organism as a whole. Consistent with the fetal tissue explant results, we also find that the epigenetic clock tracks with *in vitro* age in pluripotent stem cell-derived tissues. Stem cell-derived organoids not only recapitulated the normal developmental timing, gene expression, and organization of the fetal retina, but also progressed in the DNAm age as a function of the time from the initiation of the differentiation protocol. Interestingly, the clock seemed slightly accelerated at later stages of development compared with the age-matched fetal samples, but it still remained highly correlated with developmental age. A previous report by Horvath demonstrated that the process of generating iPS cells resets the epigenetic clock, and undifferentiated cells are significantly younger than postnatal and adult tissues^7^. While DNAm age of stem cells is negative as expected, stem cells exhibit a range of ages, between −0.2 to −0.5 years, depending on passage number and effects of different culture condition (with or feeders). The relative acceleration of the stem cell-derived retinal organoids in our experiments might therefore reflect an incomplete initial reset of the epigenetic clock in pluripotent stem cells.

Our results also have several implications for stem cell biology and disease modeling. Our data demonstrates that the epigenetic clock is a reliable and quantitative determinant of gestational age and developmental stage at fetal stages of human development, and can serve as a biomarker of accelerated aging in a progeroid syndrome^8^. Although iPSC-derived *in vitro* models of age-related diseases can sometimes demonstrate phenotypic changes related to the disease *in vitro*^14,15,41^, this often requires the cultures be stressed. The fact that the epigenetic clock tracks normal aging as early as fetal stages in a manner that is essentially equivalent to age-matched organoids, provides a biomarker for age-related diseases that may not require the manifestation of the age-related phenotype. Moreover, analyses of samples with Down syndrome show the pronounced acceleration of the DNAm age even in fetal stages, similar to what has been observed in adult tissues^33^. These findings support the conclusion that this biomarker of aging can be used from conception throughout the lifespan, for both normal and pathological aging. Our results also suggest that the mechanisms underlying the epigenetic clock (and aging more generally) might also be productively studied during development using *in vitro* cultures, either in organoids or explant cultures of fetal tissue.

## Materials and Methods

All methods were performed following NIH and University of Washington guidelines and regulations.

### Retinal explant cultures

Human fetal retinas with no identifiers were obtained from the Birth Defects Research Laboratory under an approved protocol (UW5R24HD000836) through the University of Washington. For whole retina RNAseq and DNA methylation studies, retinas were dissected and stored in Triol (Invitrogen, Carlsbad, CA) at −80C. For immunohistochemistry, whole eyes were fixed in 4% paraformaldehyde for 1 hr at room temperature, cryoprotected in a sucrose gradient, and frozen in OCT (Sakura Finetek, Torrence, CA). Gender was determined by either anatomic identification or PCR. Age was estimated by a combination of clinical intakes, gestational ultrasound, crown-rump, and fetal foot length^42,43^.

Retinas were dissected at the indicated ages and explanted onto 0.4 µm tissue culture inserts (Millipore, Billerica, MA). For the transcriptome and methylation studies, pairs of retinas were dissected and one was homogenized in Trizol immediately while the other eye was explanted and maintained in Retinal media (DMEM/F12 with GlutaMAX media containing 1% N2, 2% B27, 1% FBS, 1% PenStrep, 1x NEAA, 1x NaPyr, HEPES, and BDNF). For RNAseq studies, retinal explants were collected after 21 days in culture, and for DNA methylation assays, retinal explants were maintained for 42 days. For immunohistochemistry, unpaired retinas were cultured for 21 days.

### Immunofluorescence and microscopy

20 µm serial sections of cryoembedded retinas were first blocked in blocking buffer (10% normal horse serum in 0.5% Triton-X100/PBS) for 1 hour at room temperature and then incubated in primary antibodies diluted in blocking buffer overnight at 4C. The following day, retinas were washed 5 times in PBS, incubated with the appropriate 488/568/647 fluorophore-conjugated secondary antibodies and DAPI for 1 hour at room temperature. Samples were washed again before cover slipping using Fluoromount-G (SouthernBiotech, Birmingham, AL). Details of the antibodies used in this study are provided in Extended Data Table 2.

### Whole retina RNAseq

Total RNA from acutely collected or cultured whole retinal pairs (D59, D105, and D134) were extracted according to manufacturer’s instructions. RNA integrity was assessed using the Agilent 4200 TapeStation (Agilent Technologies, Santa Clara, CA) and quantified with Trinean DropSense Spectrophotometer (PerkinElmer). RNAseq libraries were generated using the TruSeq RNA Sample Prep kit (Illumina) and 50 base pair paired-end sequencing was performed using an Illumina HiSeq. 2500. Intersection-strict (https://htseq.readthedocs.io/en/master/count.html).

### DNA extraction and sample preparation for DNA methylation

Genomic DNA of the retinas was precipitated from the interphase and phenol phase of the TRIzol solution using 100% ethanol. The DNA pellet was washed 3 times with 0.1 M sodium citrate/10% ethanol solution and once with 75% ethanol. The pellet was then resuspended in 8 mM NaOH buffer or concentrated using gDNA Clean and Concentrator kit (Zymo Research, Irvine, CA). Concentration of gDNA was measured using the Nanodrop. Bisulfite conversion was performed on 500 ng of DNA using the llumina Infinium Methylation Assay protocol in the EZ DNA Methylation kit (Zymo Research). Following the desulphonation and wash steps, converted DNA was eluted in 12 µl of the M-Elution buffer and then loaded onto the Infinium Human Methylation 450K BeadChip kit (Illumina, #WG-314-1003) or Infinium Methylation EPIC BeadChip kit (Illumina, #WG-317-1002). Methylation dataset of the cerebral cortex was previously published^44^.

### Epigenetic clock analysis

The standard protocol of Illumina methylation assays quantifies methylation levels by the β value using the ratio of intensities between methylated (signal A) and un-methylated (signal B) alleles. Thus, β values range from 0 (completely un-methylated) to (completely methylated)^45,46^. We used the “noob” normalization method implemented in the “minfi” R package^47,48^, but our findings remain qualitatively the same for all other normalization methods implemented in the “minfi” package. A vast body of literature has demonstrated that chronological age has a profound effect on DNAm levels^49–53^. Recent studies demonstrate that DNAm levels give rise to accurate estimators of age^3,5,54^. Unlike previous age estimators that apply to saliva or blood, the pan tissue DNAm age estimator, based on 353 CpGs, is broadly applicable to all human nucleated cells, tissues, and organs. The pan tissue age estimator is defined as a prediction method of age based on the DNAm levels of 353 CpGs. The predicted age, referred to as epigenetic age or DNAm age, is in units of years. Negative DNAm age estimates indicate prenatal samples whereas positive DNAm age estimates correspond to postnatal samples. DNAm age correlates with chronological age (at the time of sample collection) in sorted cell types (glial cells, neurons, CD4+ T cells, monocytes, B cells) and tissues and organs including whole blood, brain, breast, kidney, liver, lung, and saliva^7^. The epigenetic clock (and software) applies to data generated using all Illumina array platforms (Infinium 27K, 450K, and EPIC). Mathematical details and software tutorials for the epigenetic clock can be found in the Additional files of Horvath, 2013^7^.

### Whole retina RNAseq data analysis

RNAseq data from developing human (D59–D136) retinas were generated previously^27^. Differentially expressed genes from the human dataset were reclustered using affinity propagation clustering, using the R package apcluster (v.1.4.4) and corSimMat argument. The supercluster designation (SC1–6) of a gene is the same as previously described^27^. Trisomy 21 retinas and retinal organoids (Day20–Day90) were sequenced at Fred Hutch Cancer Research Center. The quality of the FASTQ files was evaluated using FastQC (https://www.bioinformatics.babraham.ac.uk/projects/fastqc/) and reads that didn’t pass Illumina’s base call quality threshold were removed. We used HISAT v2.1.0 (https://ccb.jhu.edu/software/hisat2/index.shtml) to align reads to the hg38 reference, then counts were called using HTseq-count v0.6.0 using intersection-strict (https://htseq.readthedocs.io/en/master/count.html). Genes that had 0 counts across all samples were removed, and a table of normalized, log2-transformed counts per million (CPM) values were generated using edgeR (v.3.18.1).

### Stem cell culture

hPSCs were maintained and differentiated as described previously^16,30,32,55,56^. Briefly, cell lines H1 and H9 (WiCell, ISCRM core) were maintained in Matrigel (BD Biosciences) and mTeSR1 (Stemcell technologies) medium. Differentiation was initiated by passaging with dispase and the resultant embryoid bodies were transitioned to a 1:1 combination of TeSR and Neural induction medium (NIM; DMEM/F12, N2 supplement, MEM non-essential amino acids, Pen Strep and heparin (2 µg/ml)) for 2 days, followed by complete NIM by Day 3 onwards. On day 5, BMP4 (0.05 µg/µL, R&D systems) was added to the EBs and these were subsequently plated with 10% FBS on day 8, followed by half-medium changes every other day up to Day 12. Optic-vesicle like areas were morphologically identified by Day 20 and were manually dissected with a 30G needle attached to a 1ml syringe. These aggregates assembled into 3D spheres termed retinal organoids, and were maintained in Retinal differentiation medium (RDM; DMEM/F12, B27, MEM non-essential amino acids and Pen Strep) with 5% FBS and 2 mM Glutamax (Gibco) for long-term cultures.

### Statistics

All statistics were performed on GraphPad Prism 7/8 or in R (version 3.5.1). Two-sided paired t-tests were performed to test the differences of SC4 genes before and after culturing (Fig. 2D). ANOVA was used to calculate the slopes and the pvalues for the explant cultures (Fig. 2E). For all other tests, one-sided paired t-test was performed. *t*-tests were significant if P < 0.05.

## Supplementary information

Supplemental Figures

Extended Data Table 3

## Data Availability

Whole retina RNAseq and DNA methylation array data will be deposited in the SRA (NCBI) and Gene Expression Omnibus.
