# Supplementary material for: Synchrony and asynchrony between an epigenetic clock and developmental timing
Source: Sci Rep. 2019 Mar 6;9:3770. doi: 10.1038/s41598-019-39919-3 (PMC6403397; doi:10.1038/s41598-019-39919-3)
Supplement: Supplementary file 1 — Supplemental Figures [file 41598_2019_39919_MOESM1_ESM.pdf]

Supplementary Files

Synchrony and asynchrony between an epigenetic clock and developmental timing

Akina Hoshino[1+], Steve Horvath[2+], Akshayalakshmi Sridhar[1+], Alex Chitsazan[3], Thomas Reh[1*]

Supplementary Figures 1-5

Extended Data Tables 1 and 2

[1] Department of Biological Structure, University of Washington, Seattle, WA 98195, USA
[2] Human Genetics and Biostatistics, David Geffen School of Medicine, University of California Los Angeles, Gonda Research Center, Los Angeles, CA 90095-7088, USA.
[3]Department of Biochemistry, University of Washington, Seattle, WA 98195, USA
* Corresponding author: tomreh@uw.edu
[+] First three authors contributed equally to the work

Supplemental figure 1: Epigenetic age estimates of the cortex (Numata et al. 2012) and fetal retina. Uncalibrated DNAm age estimates show a strong non-linear relationship with chronological age, retina is shown in red and cortex in black (A-C). Calibrated DNAm age refers to the linear transformation of the uncalibrated estimates (D-F).

Supplemental figure 2: DNAm biomarkers versus age (defined as gestational age plus days in vitro) in retina samples. A) Pan tissue estimator from Horvath 2013. B) Blood based DNAm age estimator from Hannum et al. 2013[1], C) Skin and blood clock from Horvath et al. 2018[2], D) DNAm PhenoAge estimator from Levine et al. 2018[3], DNAm GrimAge estimator from Lu 2018 and F) Proportion of neurons estimated with the CETS algorithm[4]. Horvath age estimate has a high positive correlation with time (r=0.78), with negative values representative of prenatal ages less than minus 0.75 years, y-axis. Other estimators lead to less accurate prenatal age estimates (minus 20 years) or negative correlations with time.

Supplemental figure 3: A-B. Retinal organization and differentiation is conserved across D57 fetal retina (A) and D54 stem cell-derived retinal organoid (B) despite differences in size (Scale =200 µm). At this stage, the retina primarily consists of ganglion cells (ELAVL3/4, green) and progenitors (SOX9, cyan, progenitors) in the inner nuclear layer (A' and B' refers to the white box region in A and B respectively, scale = 50 µm) while photoreceptors (OTX2, magenta) are just beginning to form. Within the fetal retina, there is a central (C) to periphery (P) gradient, where the central retina is accelerated by several weeks compared to the periphery (D).

Supplemental Figure 4: A. D72 fetal retina exhibit increased photoreceptor expression from 21 days to 42 days of culture, and early bipolar expression (OTX2 in magenta and RCVRN in yellow in INL). B Explants at D103 exhibited increased RCVRN expression and expressed short wavelength S opsin proteins (SW opsin, cyan), whose morphology became more elongated as the as the ONL matured,

Supplemental figure 5: A. RNAseq comparison of WT and trisomy21 retinas revealed that chromosome 21 genes were highly upregulated in the trisomy retinas as expected. B. Total read counts between the trisomy and the control samples remained consistent. C. Scatter plot illustrating a strong correlation between the control and trisomy age-matched samples

1 Hannum, G. *et al.* Genome-wide methylation profiles reveal quantitative views of human aging rates. *Mol Cell* **49**, 359-367, doi:10.1016/j.molcel.2012.10.016 (2013).

2 Horvath, S. *et al.* Epigenetic clock for skin and blood cells applied to Hutchinson Gilford Progeria Syndrome and ex vivo studies. *Aging* **10**, 1758-1775, doi:10.18632/aging.101508 (2018).

3 Levine, M. E. *et al.* An epigenetic biomarker of aging for lifespan and healthspan. *Aging* **10**, 573-591, doi:10.18632/aging.101414 (2018).

4 Guintivano, J., Aryee, M. J. & Kaminsky, Z. A. A cell epigenotype specific model for the correction of brain cellular heterogeneity bias and its application to age, brain region and major depression. *Epigenetics* **8**, 290-302, doi:10.4161/epi.23924 (2013).

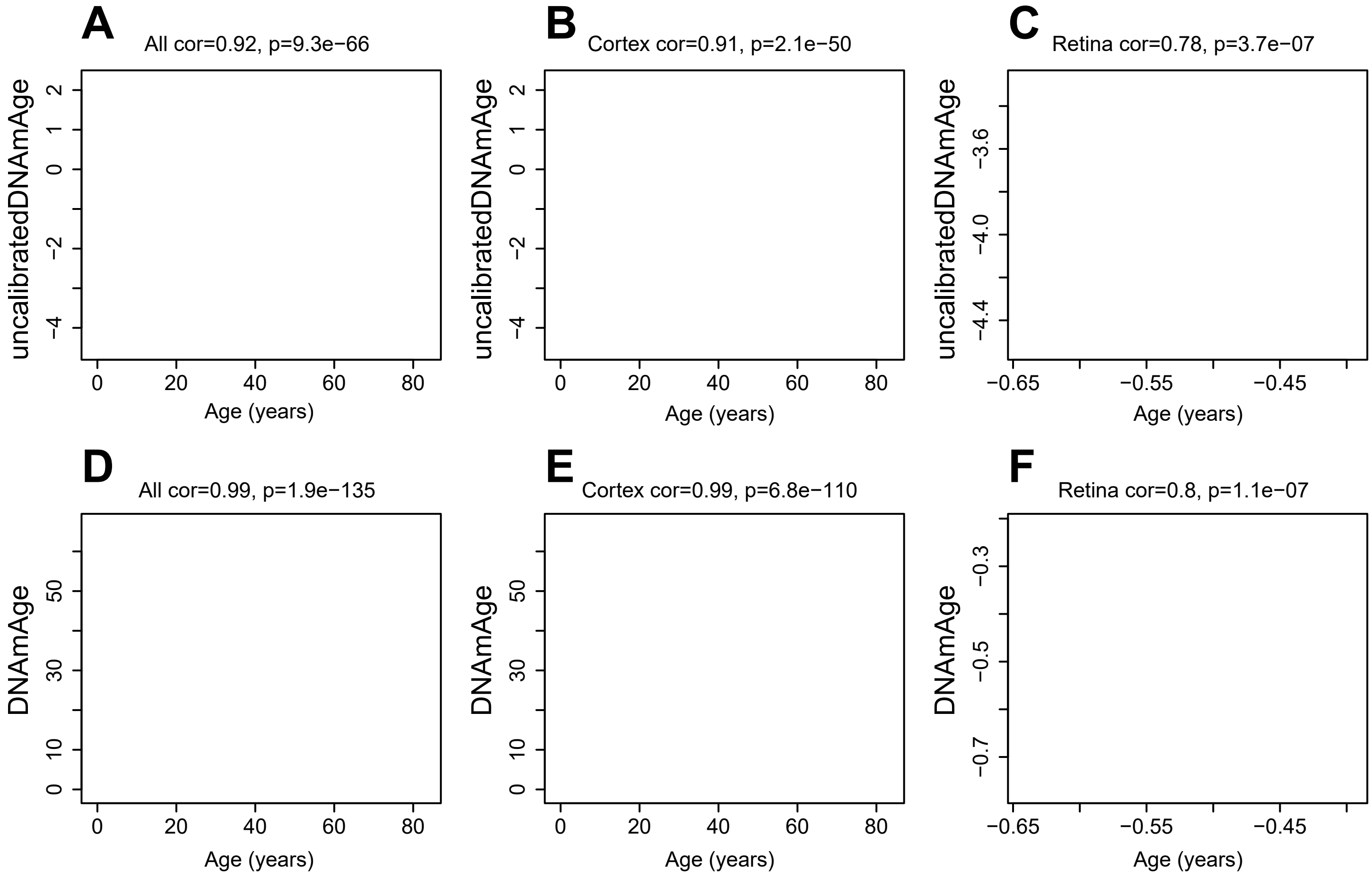

A
All cor=0.92, p=9.3e−66
uncalibratedDNAmAge
Age (years)
B
Cortex cor=0.91, p=2.1e−50
uncalibratedDNAmAge
Age (years)
C
Retina cor=0.78, p=3.7e−07
uncalibratedDNAmAge
Age (years)
D
All cor=0.99, p=1.9e−135
DNAmAge
Age (years)
E
Cortex cor=0.99, p=6.8e−110
DNAmAge
Age (years)
F
Retina cor=0.8, p=1.1e−07
DNAmAge
Age (years)

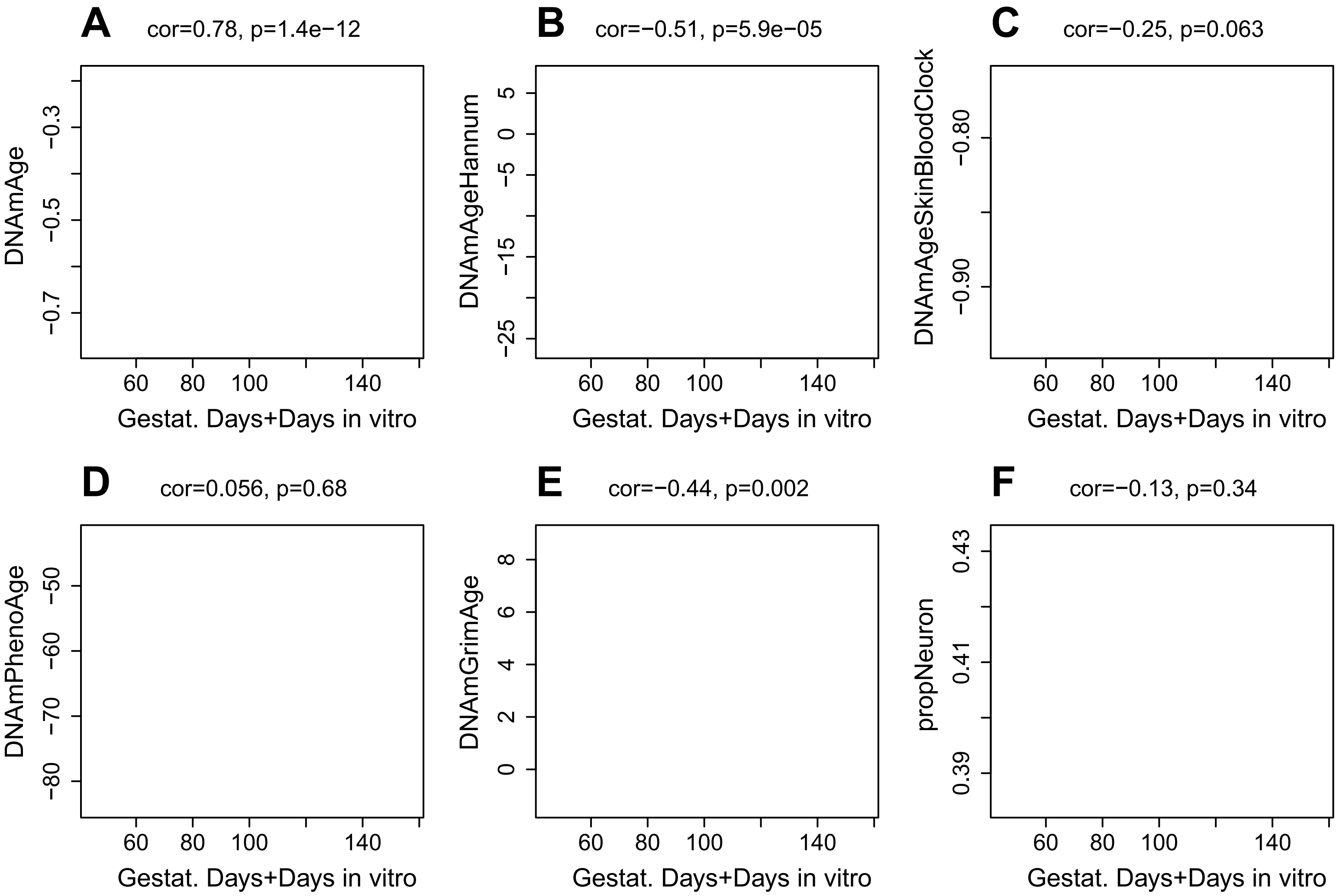
A
cor=0.78, p=1.4e−12
DNAmAge
−0.3
−0.5
−0.7
60
80
100
140
Gestat. Days+Days in vitro
B
cor=−0.51, p=5.9e−05
DNAmAgeHannum
5
0
−5
−15
−25
60
80
100
140
Gestat. Days+Days in vitro
C
cor=−0.25, p=0.063
DNAmAgeSkinBloodClock
−0.80
−0.90
60
80
100
140
Gestat. Days+Days in vitro
D
cor=0.056, p=0.68
DNAmPhenoAge
−50
−60
−70
−80
60
80
100
140
Gestat. Days+Days in vitro
E
cor=−0.44, p=0.002
DNAmGrimAge
8
6
4
2
0
60
80
100
140
Gestat. Days+Days in vitro
F
cor=−0.13, p=0.34
propNeuron
0.43
0.41
0.39
60
80
100
140
Gestat. Days+Days in vitro

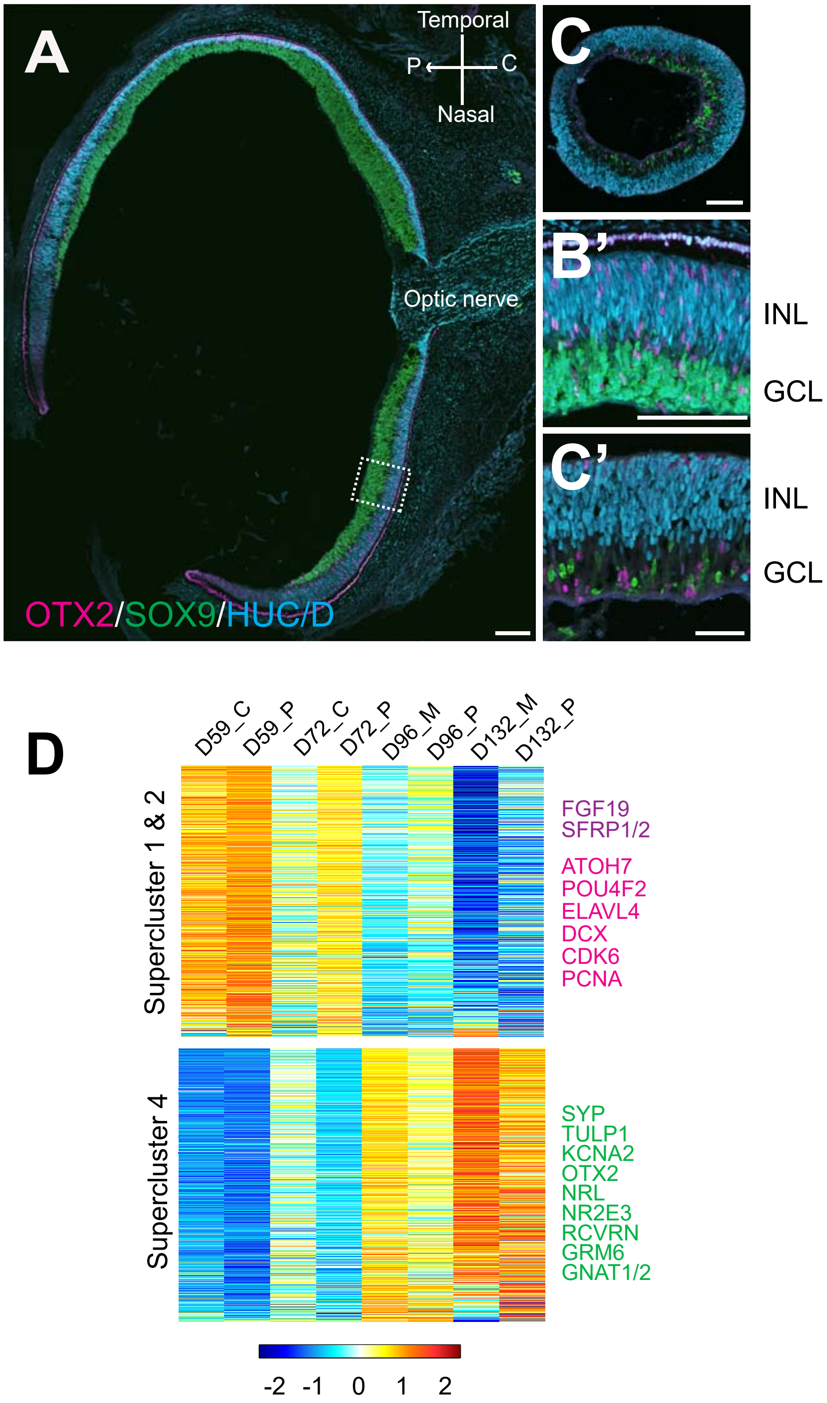

A
Temporal
P
C
Nasal
Optic nerve
OTX2/SOX9/HUC/D
C
B'
INL
GCL
C'
INL
GCL
D
D59_C
D59_P
D72_C
D72_P
D96_M
D96_P
D132_M
D132_P
Supercluster 1 & 2
FGF19
SFRP1/2
ATOH7
POU4F2
ELAVL4
DCX
CDK6
PCNA
Supercluster 4
SYP
TULP1
KCNA2
OTX2
NRL
NR2E3
RCVRN
GRM6
GNAT1/2
-2
-1
0
1
2

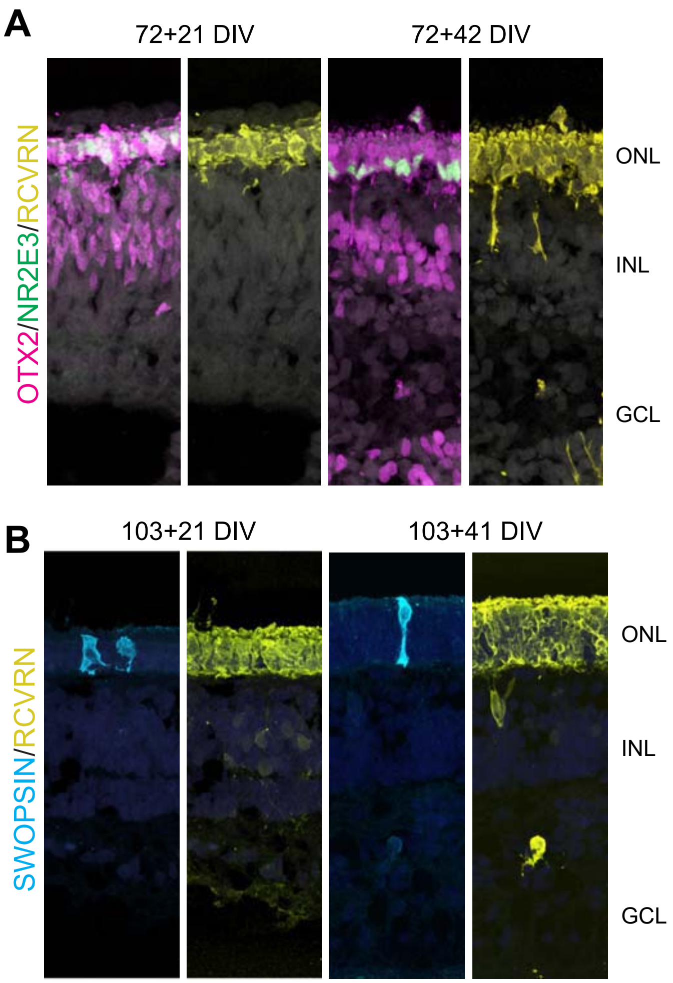
A
72+21 DIV
72+42 DIV
OTX2/NR2E3/RCVRN
ONL
INL
GCL
B
103+21 DIV
103+41 DIV
SWOPSIN/RCVRN
ONL
INL
GCL

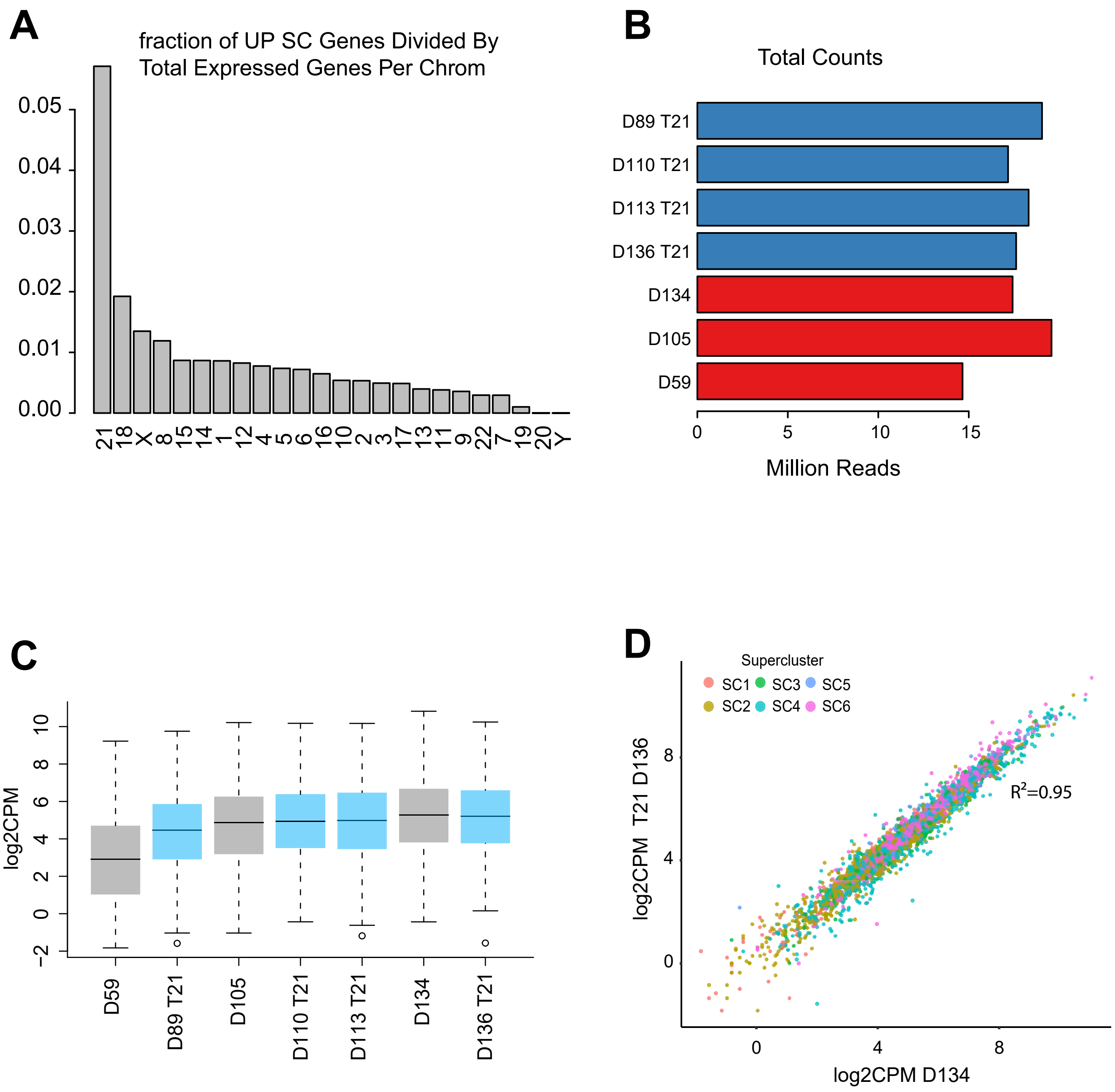
A
fraction of UP SC Genes Divided By
Total Expressed Genes Per Chrom
0.05
0.04
0.03
0.02
0.01
0.00
21 18 X 8 15 14 1 12 4 5 6 16 10 2 3 17 13 11 9 22 7 19 20 Y
B
Total Counts
D89 T21
D110 T21
D113 T21
D136 T21
D134
D105
D59
0 5 10 15
Million Reads
C
log2CPM
−2 0 2 4 6 8 10
D59
D89 T21
D105
D110 T21
D113 T21
D134
D136 T21
D
Supercluster
SC1 SC3 SC5
SC2 SC4 SC6
log2CPM T21 D136
0 4 8
R2=0.95
0 4 8
log2CPM D134

Extended Data Table 1: Timing of retinal differentiation in mouse and human pluripotent stem cells

Mouse:

**Stages of retinal development**	**Differentiation day**	**References**
Eye field	D5	[1]
Optic vesicle	D5–D8;	[1-9]
Optic cup	D7–13	[1-3,7]

**Cell-specific markers**	**Differentiation day**	**References**
Progenitors	D7–12 (Rax, Pax6, Vsx2, EdU)	[1,5,6,9]
Ganglion cells	D9 (Pou4f2, Islet, Elavl3/4, Tuj1, Pax6)	[1,2,4-7,9,10]
Horizontal/Amacrine cells	D14–26 (Calb1, Calb2, Pax6, Prox1)	[2,3,5,7,10]
Cone photoreceptors	Early markers:<br>D12–16 (Crx, Rxrg)<br>Late markers:<br>D22–29 (Opn1sw, Opn1mw/Opn1lw)	[1,3,4,6,9,10]
Rod photoreceptors	Early markers:<br>D15–21 (Crx, Nrl, Rcvrn)<br>Late markers:	[1-3,5-11]

	D24–28 (Rho)	
Bipolar cells	D24–32 (Otx2+/Crx-, Prkca, Vsx2)	[2-4,7,9,10]
Müller glia	D25–28 (Rlbp1, Vim, Glul)	[3-5,7,9]
Synaptic markers	D22–32 (Bsn, CtBP2, Syp)	[4,7,9]

Human:

**Stages of retinal development**	**Differentiation day**	**References**
Eye field	Week 1–2 (RAX, PAX6, SIX3, LHX2, SIX6)	[12-15]
Optic vesicle	Week 2	[16]
Optic cup	Week 3	[16]

**Cell type**	**Differentiation day**	**References**
Progenitors	Week 2–6 (PAX6, RAX, VSX2, SOX2, SOX9)	[6,10,12-14,16-20]
Ganglion cells	Week 3–9	[14-19,21-26]
Horizontal and amacrine cells	Week 6–18 (PTF1A, CALB1, CALB2, PROX1, TFAP2, ELAVL3/4)	[14,16,17,19,20,23,26]

Cone and rod photoreceptors	Pan photoreceptor markers:<br><br>Week 3–24 (CRX, OTX2, RCVRN, AIPL1, PRDM1<br><br>Early cone markers<br><br>WK7 -19 (RXRG, ONECUT1, ARR3)<br><br>Late cone markers<br><br>Week 11–24 (OPN1MW/OPN1LW, OPN1SW, PDE6C, ARR3)<br><br>Early rod markers<br><br>Week 9–18 (NRL, NR2E3)<br><br>Late rod markers<br><br>Week 15–29 (RHO, PDE6B, CNGA1, GNAT1, SAG)	[10,12,14-28]
Bipolar cells	WK9–23 (PRKCA, VSX2/MCM2-, CABP5, GRM6)	[19,26]
Müller glia	Week 12–17 (GLUL/SOX9, RLBP1)	[17,19,23]
Synapses	W12–23 (SV2, SLC17A7, BSN, CTBP2)	[19,22,29]

1 La Torre, A., Hoshino, A., Cavanaugh, C., Ware, C. B. & Reh, T. A. The GIPC1-Akt1 Pathway Is Required for the Specification of the Eye Field in Mouse Embryonic Stem Cells. *Stem Cells* **33**, 2674-2685, doi:10.1002/stem.2062 (2015).

2 Eiraku, M. *et al.* Self-organizing optic-cup morphogenesis in three-dimensional culture. *Nature* **472**, 51-56, doi:10.1038/nature09941 (2011).

3 Gonzalez-Cordero, A. *et al.* Photoreceptor precursors derived from three-dimensional embryonic stem cell cultures integrate and mature within adult degenerate retina. *Nat Biotechnol* **31**, 741-747, doi:10.1038/nbt.2643 (2013).

4 Decembrini, S., Koch, U., Radtke, F., Moulin, A. & Arsenijevic, Y. Derivation of traceable and transplantable photoreceptors from mouse embryonic stem cells. *Stem Cell Reports* **2**, 853-865, doi:10.1016/j.stemcr.2014.04.010 (2014).

5 Assawachananont, J. *et al.* Transplantation of embryonic and induced pluripotent stem cell-derived 3D retinal sheets into retinal degenerative mice. *Stem Cell Reports* **2**, 662-674, doi:10.1016/j.stemcr.2014.03.011 (2014).

6 Volkner, M. *et al.* Retinal Organoids from Pluripotent Stem Cells Efficiently Recapitulate Retinogenesis. *Stem Cell Reports* **6**, 525-538, doi:10.1016/j.stemcr.2016.03.001 (2016).

7 Chen, H. Y., Kaya, K. D., Dong, L. & Swaroop, A. Three-dimensional retinal organoids from mouse pluripotent stem cells mimic in vivo development with enhanced stratification and rod photoreceptor differentiation. *Mol Vis* **22**, 1077-1094 (2016).

8 Hiler, D. *et al.* Quantification of Retinogenesis in 3D Cultures Reveals Epigenetic Memory and Higher Efficiency in iPSCs Derived from Rod Photoreceptors. *Cell stem cell* **17**, 101-115, doi:10.1016/j.stem.2015.05.015 (2015).

9 DiStefano, T. *et al.* Accelerated and Improved Differentiation of Retinal Organoids from Pluripotent Stem Cells in Rotating-Wall Vessel Bioreactors. *Stem Cell Reports* **10**, 300-313, doi:10.1016/j.stemcr.2017.11.001 (2018).

10 Osakada, F. *et al.* Toward the generation of rod and cone photoreceptors from mouse, monkey and human embryonic stem cells. *Nat Biotechnol* **26**, 215-224, doi:10.1038/nbt1384 (2008).

11 Homma, K. *et al.* Developing rods transplanted into the degenerating retina of Crx-knockout mice exhibit neural activity similar to native photoreceptors. *Stem Cells* **31**, 1149-1159, doi:10.1002/stem.1372 (2013).

12 Meyer, J. S. *et al.* Modeling early retinal development with human embryonic and induced pluripotent stem cells. *Proc Natl Acad Sci U S A* **106**, 16698-16703, doi:10.1073/pnas.0905245106 (2009).

13 Lamba, D. A., Karl, M. O., Ware, C. B. & Reh, T. A. Efficient generation of retinal progenitor cells from human embryonic stem cells. *Proc Natl Acad Sci U S A* **103**, 12769-12774, doi:10.1073/pnas.0601990103 (2006).

14 Lamba, D. A. *et al.* Generation, purification and transplantation of photoreceptors derived from human induced pluripotent stem cells. *PLoS One* **5**, e8763, doi:10.1371/journal.pone.0008763 (2010).

15 Phillips, M. J. *et al.* Blood-derived human iPS cells generate optic vesicle-like structures with the capacity to form retinal laminae and develop synapses. *Invest Ophthalmol Vis Sci* **53**, 2007-2019, doi:10.1167/iovs.11-9313 (2012).

16 Nakano, T. *et al.* Self-formation of optic cups and storable stratified neural retina from human ESCs. *Cell stem cell* **10**, 771-785, doi:10.1016/j.stem.2012.05.009 (2012).

17 Reichman, S. *et al.* From confluent human iPS cells to self-forming neural retina and retinal pigmented epithelium. *Proc Natl Acad Sci U S A* **111**, 8518-8523, doi:10.1073/pnas.1324212111 (2014).

18 Ohlemacher, S. K. *et al.* Stepwise Differentiation of Retinal Ganglion Cells from Human Pluripotent Stem Cells Enables Analysis of Glaucomatous Neurodegeneration. *Stem Cells* **34**, 1553-1562, doi:10.1002/stem.2356 (2016).
19 Zhong, X. *et al.* Generation of three-dimensional retinal tissue with functional photoreceptors from human iPSCs. *Nat Commun* **5**, 4047, doi:10.1038/ncomms5047 (2014).
20 Meyer, J. S. *et al.* Optic vesicle-like structures derived from human pluripotent stem cells facilitate a customized approach to retinal disease treatment. *Stem Cells* **29**, 1206-1218, doi:10.1002/stem.674 (2011).
21 Zhu, J. *et al.* Generation of Transplantable Retinal Photoreceptors from a Current Good Manufacturing Practice-Manufactured Human Induced Pluripotent Stem Cell Line. *Stem Cells Transl Med* **7**, 210-219, doi:10.1002/sctm.17-0205 (2018).
22 Wahlin, K. J. *et al.* Photoreceptor Outer Segment-like Structures in Long-Term 3D Retinas from Human Pluripotent Stem Cells. *Sci Rep* **7**, 766, doi:10.1038/s41598-017-00774-9 (2017).
23 Kaewkhaw, R. *et al.* Transcriptome Dynamics of Developing Photoreceptors in Three-Dimensional Retina Cultures Recapitulates Temporal Sequence of Human Cone and Rod Differentiation Revealing Cell Surface Markers and Gene Networks. *Stem Cells* **33**, 3504-3518, doi:10.1002/stem.2122 (2015).
24 Browne, A. W. *et al.* Structural and Functional Characterization of Human Stem-Cell-Derived Retinal Organoids by Live Imaging. *Invest Ophthalmol Vis Sci* **58**, 3311-3318, doi:10.1167/iovs.16-20796 (2017).
25 Vergara, M. N. *et al.* Three-dimensional automated reporter quantification (3D-ARQ) technology enables quantitative screening in retinal organoids. *Development* **144**, 3698-3705, doi:10.1242/dev.146290 (2017).
26 Phillips, M. J. *et al.* A Novel Approach to Single Cell RNA-Sequence Analysis Facilitates In Silico Gene Reporting of Human Pluripotent Stem Cell-Derived Retinal Cell Types. *Stem Cells*, doi:10.1002/stem.2755 (2017).
27 Welby, E. *et al.* Isolation and Comparative Transcriptome Analysis of Human Fetal and iPSC-Derived Cone Photoreceptor Cells. *Stem Cell Reports* **9**, 1898-1915, doi:10.1016/j.stemcr.2017.10.018 (2017).
28 Singh, R. K. *et al.* Characterization of Three-Dimensional Retinal Tissue Derived from Human Embryonic Stem Cells in Adherent Monolayer Cultures. *Stem Cells Dev* **24**, 2778-2795, doi:10.1089/scd.2015.0144 (2015).
29 Mellough, C. B. *et al.* IGF-1 Signaling Plays an Important Role in the Formation of Three-Dimensional Laminated Neural Retina and Other Ocular Structures From Human Embryonic Stem Cells. *Stem Cells* **33**, 2416-2430, doi:10.1002/stem.2023 (2015).

Extended Data Table 2: Antibodies used in this study.

Antibody	Source	Catalog #	Dilution
CALBINDIN	Swant Swiss antibodies	CD38	1:250
ELAVL3/4	Invitrogen	A-21271	1:200
NR2E3	Abcam	Ab172542	1:250
OTX2	R&D Systems	BAF1979	1:200
POU4F2	Santa Cruz Biotechnology	SC-6026	1:250
RCVRN	Chemicon	AB5585	1:2000
SOX2	Santa Cruz	sc-17320	1:100
VSX2	Santa Cruz	SC-21690	1:300
VGLUT1	Synaptic systems	135 304	1:500
Donkey anti-goat 488	Life Technologies	A11055	1:250
Donkey anti-goat 568	Life Technologies	A11057	1:250
Donkey anti-mouse 488	Jackson Immuno	715-546-151	1:250
Donkey anti-mouse 568	Life Technologies	A10037	1:250
Donkey anti-rabbit 647	Thermo Fisher Scientific	A-31573	1:250
DAPI	Sigma	D9542	1:5000
